# Predictors of Health-Related Quality of Life in Neurodivergent Children: A Systematic Review

**DOI:** 10.1007/s10567-023-00462-3

**Published:** 2023-12-09

**Authors:** Maryam Mahjoob, Tithi Paul, Julia Carbone, Harshit Bokadia, Robyn E. Cardy, Souraiya Kassam, Evdokia Anagnostou, Brendan F. Andrade, Melanie Penner, Azadeh Kushki

**Affiliations:** 1 University of Toronto, Toronto, ON Canada; 2 Bloorview Research Institute, Holland Bloorview Kids Rehabilitation Hospital, 150 Kilgour Road, Toronto, ON M4G 1R8 Canada; 3 Margaret and Wallace McCain Centre for Child Youth and Family Mental Health, Centre for Addiction and Mental Health, Toronto, ON Canada; 4 Department of Psychiatry, University of Toronto, Toronto, ON Canada

**Keywords:** Health-related quality of life, Neurodevelopmental conditions

## Abstract

Health-related Quality of Life (HRQoL) is a multi-faceted construct influenced by a myriad of environmental, demographic, and individual characteristics. Our understanding of these influencers remains highly limited in neurodevelopmental conditions. Existing research in this area is sparse, highly siloed by diagnosis labels, and focused on symptoms. This review synthesized the evidence in this area using a multi-dimensional model of HRQoL and trans-diagnostically across neurodevelopmental conditions. The systematic review, conducted in accordance with the Preferred Reporting Items for Systematic Reviews and Meta-Analysis Checklist, was completed in June 2023 using Medline, PsycInfo, Embase, PubMed, and Cochrane Library. Our search revealed 78 studies that examined predictors of HRQoL in neurodevelopmental conditions. The majority of these studies focused on autism and ADHD with a paucity of literature in other conditions. Cross-diagnosis investigations were limited despite the fact that many of the examined predictors transcend diagnostic boundaries. Significant gaps were revealed in domains of biology/physiology, functioning, health perceptions, and environmental factors. Very preliminary evidence suggested potentially shared predictors of HRQoL across conditions including positive associations between HRQoL and adaptive functioning, male sex/gender, positive self-perception, physical activity, resources, and positive family context, and negative associations with diagnostic features and mental health symptoms. Studies of transdiagnostic predictors across neurodevelopmental conditions are critically needed to enable care models that address shared needs of neurodivergent individuals beyond diagnostic boundaries. Further understanding of HRQoL from the perspective of neurodivergent communities is a critical area of future work.

**Supplementary Information:**

The online version contains supplementary material available at 10.1007/s10567-023-00462-3.

## Background

Neurodevelopmental conditions refer to a group of heterogeneous attributes that manifest early in life and can be associated with differences and disability in personal, social, occupational, or academic functioning (“Neurodevelopmental Disorders”, 2013). These conditions include autism spectrum disorder (autism^1^; prevalence 1 in 66; Ofner et al., 2018), attention-deficit/hyperactivity disorder (ADHD; prevalence 1 in 20; Polanczyk et al., 2014), intellectual disability (ID; prevalence up to 63 in 1000), communication disorders (prevalence up to 1 in 10), learning disorders, including impairments in reading, writing and mathematics (LD; prevalence up to 1 in 10), and motor disorders (including tic disorders, and stereotypic disorders; prevalence up to 17 in 100) (Francés et al., 2022). Considerably large within-condition heterogeneity and cross-condition overlap exist in aetiology, neurobiology, and phenotypes associated with neurodevelopmental conditions (Anholt et al., 2010; Antshel et al., 2013; Astle et al., 2021; Kushki et al., 2019). These conditions can also be associated with transdiagnostic challenges that can further increase the heterogeneity of presentation and outcomes (e.g. mental health conditions (DeFilippis, 2018; Moritz, 2008; Schatz & Rostain, 2006), sleep difficulties (Díaz-Román et al., 2015, 2018), and differences in learning (DuPaul et al., 2004; Estes et al., 2011; Fischer-Terworth, 2013), and motor skills (Abramovitch et al., 2011; Damme et al., 2015). These differences and disabilities, combined with societal barriers, can lead to decreased quality of life (QoL); (Becker et al., 2011; Coales et al., 2019; Kuhlthau et al., 2010; Lack et al., 2009; Lin, 2019; Wanni Arachchige Dona et al., 2023), as one’s satisfaction in relation to their culture, value systems, goals, expectations, standards, and concerns (World Health Organization, Division of Mental Health and Prevention of Substance Abuse 2012). Further narrowing this definition, Health-Related Quality of Life (HRQoL) reflects QoL in the context of an individual’s health status, excluding the non-health-related categories such as cultural or political measurements (Ferrans et al., 2005).

Mirroring the diversity in neurodevelopmental conditions, HRQoL outcomes are highly variable in these conditions. In this context, several studies have attempted to characterize predictors of HRQoL in neurodivergent individuals. Among these, diagnostic clinical features of neurodevelopmental conditions, including features associated with autism (Ayres et al., 2018; Lin 2019), and ADHD (Danckaerts et al., 2010), have been suggested to be correlates of HRQoL. Mental health symptoms have also been associated with decreased quality of life across neurodevelopmental conditions (Lawson et al., 2020; Lin, 2019; Mason et al., 2018; Orm et al., 2023). To our knowledge, no reviews exist on transdiagnostic predictors of HRQoL in neurodevelopmental conditions, and none within the last five years on HRQoL predictors in individual diagnoses (Agarwal et al., 2012; Ayres et al., 2018; Chiang & Wineman, 2014; Danckaerts et al., 2010). A recent review is critically needed given the emerging interest in this area as demonstrated by several recent publications on predictors of HRQoL in neurodevelopmental conditions. Further, individual studies of HRQoL are almost entirely conducted in diagnostic siloes, and very little is known about transdiagnostic predictors of HrQoL in neurodevelopmental conditions. This transdiagnostic approach is critically needed in the light of the growing concern that our existing, discrete, diagnostic categories do not adequately capture experiences, align with underlying biological mechanisms, or guide the choice of supports (Anholt et al., 2010; Antshel et al., 2013; Astle et al., 2021; Kushki et al., 2019). To address this gap, the objective of the present study was to characterize the state of the literature on transdiagnostic predictors of HRQoL in neurodevelopmental conditions and generate hypotheses for future research in this area.

**Fig1:**
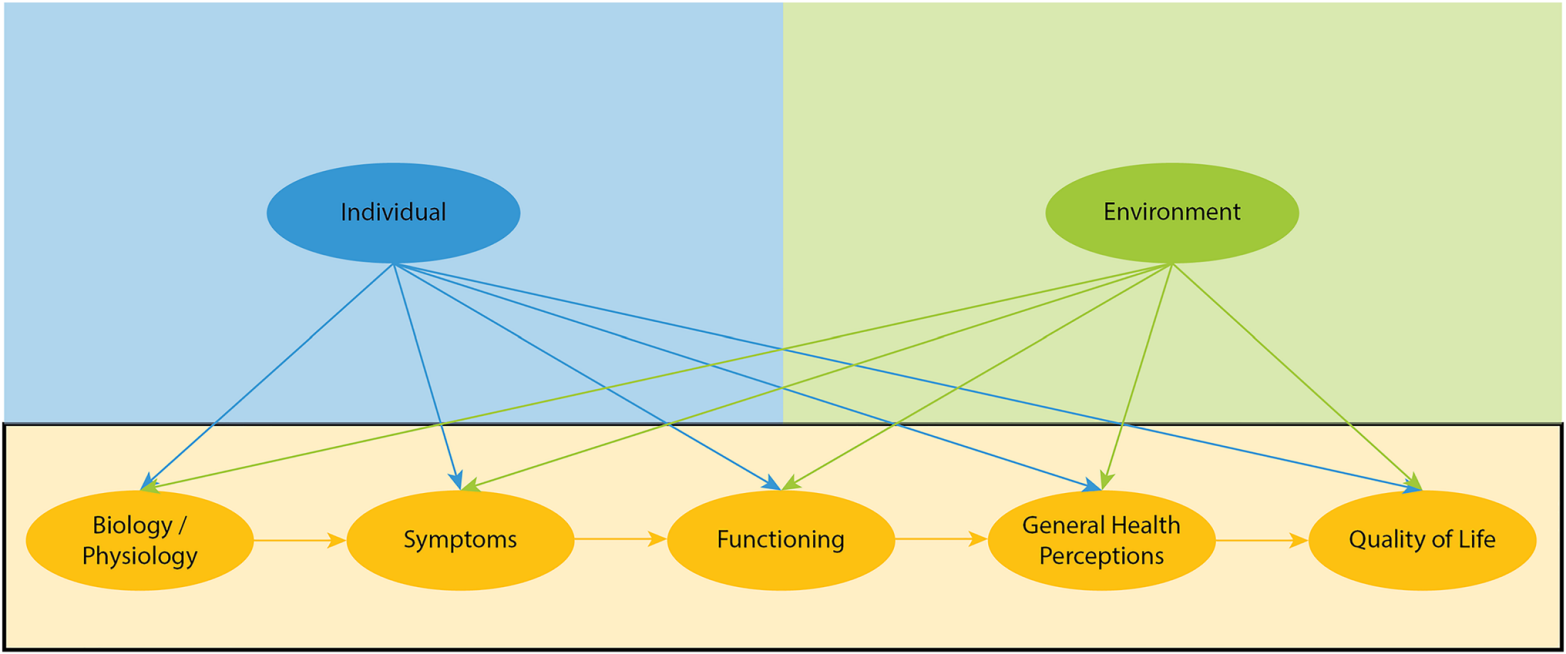
Adapted revised Wilson and Cleary Model of HRQoL by Ferrans et al.

HRQoL is a multi-dimensional and interconnected construct which can be influenced by a multitude of biological, phenotypic, environmental, and sociodemographic variables. To reflect this, we grounded our review in the theoretical framework of Wilson and Cleary, a conceptual model which links HRQoL to biological and psychosocial variables (Wilson & Cleary, 1995). For this review, we used Ferrans et al.’s revised Wilson and Cleary Model of HRQoL predictors (Ferrans et al., 2005; Fig. 1). In this model, HRQoL is impacted by four domains: (1) biological and physiological factors (functioning of one’s human body on a cellular, organ, or organ system level), (2) symptoms (physical or mental features of the human body as a whole), (3) functioning (an individual’s ability to complete physical, social, or psychological tasks), and (4) general health perceptions (the subjective feeling of health). Each of these domains is impacted by characteristics of the individual and the environment (Wilson & Cleary, 1995). Individual factors in this model include demographic group (e.g. sex, gender, age, ethnicity), biological features (e.g. body mass index, skin colour, family medical history), and psychological characteristics (e.g. cognitive appraisal, affective response, motivation; Ferrans et al., 2005). Environmental characteristics include social factors (e.g. influence of family, friends, and healthcare providers), and physical factors (e.g. neighbourhood and school; Ferrans et al., 2005). Given this theoretical grounding, our specific research question for this review was: across neurodevelopmental conditions, what are the transdiagnostic predictors of HRQoL within the domains of the revised Wilson and Cleary model?

## Methods

This systematic review protocol was designed and conducted in accordance with the Preferred Reporting Items for Systematic Reviews and Meta-Analysis Checklist (Moher et al., 2009). The full review protocol is provided in the Supplementary Materials and was registered in PROSPERO (Reg. No. CRD42023431150). Ethics approval was not needed as this review used previously completed studies. There were no published systematic reviews on this topic in the Cochrane library or PROSPERO at the time the review was designed.

### Search Strategy

Five databases were used for the search: Medline, PsycInfo, Embase, PubMed, and Cochrane. The search terms included neurodevelopmental disorders as defined in the Diagnostic and Statistical Manual of Mental disorders (DSM-5; autism/ASD, attention-deficit/hyperactivity disorder/ADHD, intellectual disorder, intellectual disability, intellectual developmental disorder, global developmental delay, communication disorder, language disorder, speech disorder, speech sound disorder, fluency disorder, stutter, learning disorder, impairment in reading, impairment in written expression, impairment in mathematics, motor disorder, developmental coordination disorder, stereotypic movement disorder, tic disorder, or Tourette), quality of life/QoL, and predict/determinant (see detailed list in Supplementary Table 1). The search was completed on 23 June 2022.

All articles were imported to Covidence to undergo screening, review, and extraction by the authors (screening and extraction: MM, TP, HB, JC; full-text review: TP, HB, JC). Inter-rater screening reliability was determined on a subset of 300 articles with the goal of greater than 80% consensus among all reviewers. For title and abstract screening, each study was assessed by two reviewers and disagreements resolved through deliberation.

### Inclusion and Exclusion Criteria

Our inclusion criteria were the following: (1) primary peer-reviewed literature published in English, (2) employed a validated measure of HRQoL in populations with neurodevelopmental disorders as defined in the DSM-5, and (3) statistically examined the association between a predictor(s) variable and a total HRQoL score. Studies that employed qualitative methods were excluded, as they did not provide a statistical quantification of the effect of a predictor on HrQoL. Theses/dissertations, conference/poster abstracts, and randomized control trials were excluded.

### Data Extraction and Analysis

Data were extracted using personalized extraction templates on Covidence (*Covidence Systematic Review Software, Veritas Health Innovation, Melbourne, Australia.*, n.d.). The extracted data included the following: title, year, HRQoL outcome measure, informant (self or proxy), country, and sample characteristics (total sample size, diagnosis, gender, age, family/self-income, parental/self-education, socioeconomic status, and race/ethnicity). Other data extracted included analysis methods, significant/non-significant predictors of HRQoL, and the associated statistics. For data extraction, one reviewer extracted the data, and a second reviewer cross-checked the extracted data. Due to the heterogeneity of the study designs, a narrative synthesis of the results took place. Risk of bias assessment was completed using an adapted Cochrane template since the review included more than one study design (see Supplementary Table 2).

The Revised Wilson and Cleary model of HRQoL predictors (Fig. 1) guided the synthesis of predictor variables. Predictors were categorized under the main domains of the model (biology/physiology, symptoms, functioning, general health perceptions), or the external domains (environmental and individual characteristics) through consensus among co-authors (Supplementary Table 3). Each domain was operationalized as follows:

- Biology/Physiology: variables measuring functioning of cells, organs, or organ systems.
- Symptoms: core-domain features of neurodevelopmental conditions as well as co-occurring symptoms in domains of behaviour and mental health. Predictors related to physical health and health care needs were also included in this category.
- Functioning: operationalized as adaptive functioning or the ability to complete demands of everyday life.
- General Health Perceptions: predictors related to the subjective feeling of health.
- Individual characteristics: variables related to demographics, psychological characteristics, healthfulness behaviours, and birth-related and anthropometric variables.
- Environmental Characteristics: birth/prenatal characteristics, parental/sibling characteristics, social and physical environment,, and access to healthcare resources.

## Results

### Literature Search

The search revealed 4025 articles after duplicates were removed. For abstract and title screening, the per cent agreement between all 3 reviewers was 81%. Title and abstract screening deemed 3582 studies as irrelevant. The most common reasons for exclusion were as follows: 1. study did not include a neurodivergent population, 2. study did not assess HRQoL, and 3. study was a review or meta-analysis.

**Fig. 2:**
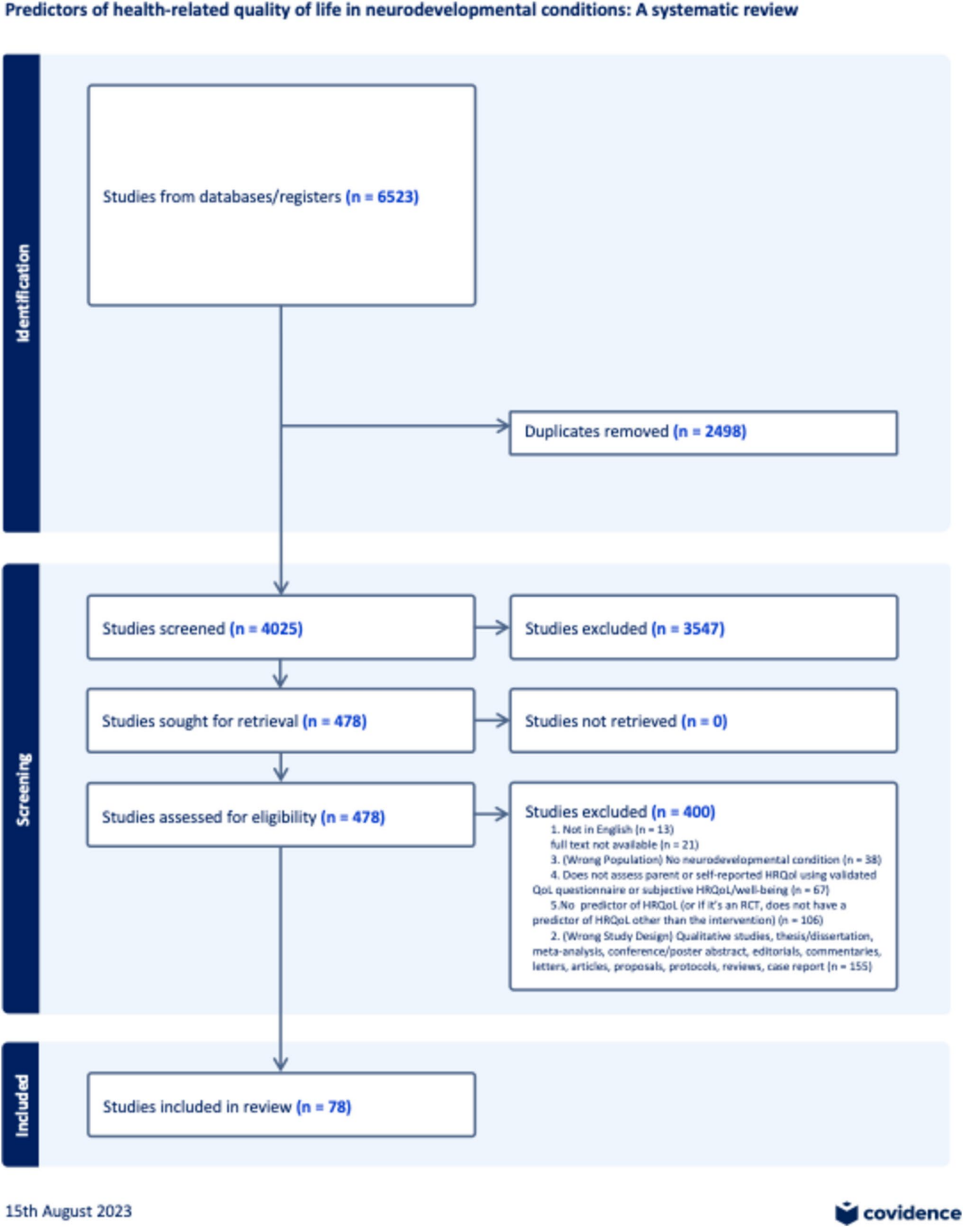
PRISMA diagram

Following this, 478 full-text studies were assessed for eligibility. For full-text review, the agreement between the reviewers was 87.8%. Upon full-text review, studies were removed due to non-English language (*n* = 13), study population not including a neurodevelopmental condition (*n* = 38), absence of total HRQoL assessment (*n* = 65), no predictors of HRQoL (*n* = 94), and study designs not meeting inclusion criteria (*n* = 155; qualitative studies, thesis/dissertations, reviews, conference/poster abstract, editorial, commentary, letter, proposals, protocols, and case reports). After these exclusions, 78 studies were included in the review as shown in the PRISMA diagram in Fig. 2.

### Study Characteristics

Of the studies included in the review, the majority (*n* = 71) had a low risk of bias, with only six and one studies with medium and high risks of bias, respectively. The most frequently identified sources of biases included sample selection and description, description of statistical methods, and reporting of statistical results. Table 1 provides the details of the reviewed studies.

**Table 1:** Detailed characteristics of the reviewed studies

Study	Country	Instrument	Informant	N	Diagnosis	Sex/gender	Age	Analysis	Domains	Bias
Adams (2019)	AUS	PedsQL	Self	71	autism	58:0:0:13	M = 10.7, SD = 2.3	Pearson Correlation, Linear regression	Symptoms: anxiety*, autism symptoms/traits Individual: age	low
Adams (2020)	AUS	PedsQL	Parent	64	autism	46:0:0:18	M = 10.1, SD = 3.1	ANOVA	Symptom: anxiety*	low
Ahnemark (2018)	Sweden	EQ5D	Self	189	ADHD	82:107:0:0	M = 33.7, SD = 12.4	Linear Regression	Symptoms: autism symptoms/traits*, ADHD symptoms, anxiety*, depression*, psychological comorbidities*Individual: age, gender/sex*, employment status*, IQ Environmental: SES*, having at least one child	low
Albuquerque (2012)	Portugal	QoL-Q	Self	78	ID	40:38:0:0	M = 25.1, SD = 7.6	Correlation	Individual: positive self-perception*	Low
Balboni (2020)	Italy	Personal Outcomes Scale Self-Report and Report of Others	Both	93	ID	43:0:0:50	M = 41.6, SD = 12.2	Hierarchical Regression	Symptoms: behavioural problems, Functioning: adaptive functioning*Individual: age, gender/sex, employment status	low
Ben-DorCohen (2021)	Israel	AAQoL	Self	63	ADHD	30:33:0:0	M = 24.9, SD = 3.3	ANOVA, Moderation analysis	Symptoms: ADHD symptoms*, emotional dysregulation*, medication	low
Bernard (2009)	US	TNO-AZL	Parent	56	Tic disorders	52:4:0:0	M = 10.5, SD = 2.9	Spearman Correlation, Multiple Regression Model	Symptoms: Tic disorder symptoms, ADHD symptoms*, years since diagnosis, obsessive–compulsive symptoms*Individual: age	low
Boyle, (2015)	US	Quality of Life Enjoyment and Satisfaction Questionnaire‚ Short Form	Self	249	stutter	Not reported	M = 40.2, SD = 15.8	Correlation	Symptoms: stutter symptoms*General health: symptom/illness identity*Individual: age*, gender/sex, empowerment*, involvement in treatment*, positive self-perception*Environmental: social support*, self-help support group/self-help organizations*	low
Capal (2020)	US; CAN	PedsQL	Self	472	autism	388:84:0:0	M = 9.6, SD = 3.2	t-test	Symptoms: seizures*	low
Caron (2022)	CAN; France	ASQoL	Self	430	autism	99:242:0:89	M = 37.0, SD = 11.1 M = 33.5, SD = 11.7	ANCOVA	Biology/physiology: physical health/well-being*Symptoms: autism symptoms/traits*, ADHD symptoms, learning disability symptoms, sensory disorder*, anxiety*, mood disorders*, medication, Individual: age*, gender/sex*, race/ethnicity, employment status*, Environmental: SES*, violence history*, age of diagnosis*	low
Carter (2017)	US; AUS	OASES-A	Self	39	stutter	31:8:0:0	M = 42.2, SD = 16.9	Pearson correlation	Symptoms: stutter symptoms, Individual: age*, self-efficacy for verbal communication*	low
Cavanna (2012)	UK	GTS-QOL	Self	46	Tic disorders	41:5:0:0	M = 10.8, SD = 3.6	Pearson correlation coefficient, independent sample t-test	Symptoms: tic disorder symptoms*, ADHD symptoms, obsessive–compulsive symptoms, self-injurious behaviour, Environmental: Family history of tics*	low
Chou (2007)	Taiwan	CCQOLI	Self	233	ID	145:88:0:0	M = 27.6, SD = 11.1	Stepwise regression	Functioning: activities of daily living*, Individual: age*, gender/sex, employment status*, Environmental: SES*, geography*	low
Corbera (2021)	US	QLS	Self	30	autism	23:7:0:0	M = 21.7, SD = 3.0	Hierarchical multiple regression	Symptoms: autism symptoms/traits*, Individual: IQ*	low
Cramm (2012)	Netherlands	ID-QOL-24	Parent	108	ID	0:41:0:67	M = 11.6, SD = 6.4	Regression	Biology/ physiology: physical health/well-being, Symptoms: depression*, Functioning: activities of daily living, Environmental: SES, social support, parental mental health*	low
Crawford (2015)	UK	Life Experience Checklist	Self	101	ID	57:44:0:0	M = 35.1, SD = 14.0	Correlation	Symptoms: anxiety, Individual: age, IQ, Environmental: social supports*	med
de Vries (2018)	Netherland	PedsQL		101	autism	Not reported	Range = 8–12	Regression	Symptoms: autism symptoms/traits*, executive functioning, Individual: IQ, reward sensitivity*	low
Dijkhuis (2017)	Netherlands	QoL-Q	Self	75	autism	67:0:0:8	M = 21.9, SD = 2.3	Hierarchical Regression	Symptoms: executive functioning*, Individual: age*, gender/sex, emotion processing	low
Doja (2018)	CAN	PedsQL	Self	13	Tic disorders	8:5:0:0	Range = grade 2 -11	Mann- Whitney U test	Individual: physical activity*	low
Dolgun (2014)	Turkey	ADHD/QoLS	Self	70	ADHD	57:0:0:13	M = 9.8, SD = 1.0	Correlation	Individual: feeling of freedom from worries/ feeling bad/ peer rejection*, positive self-evaluation in academics*, positive self-perception	low
Eapen (2016)	AUS	TS-QoL	Both	83	Tic disorders	61:0:0:22	M = 26.0	Multiple Regression, correlation	Symptoms: tic disorder symptoms*, ADHD symptoms*, psychological comorbidities*	low
Eddy (2011)	UK	YQOL-R	Self	50	Tic disorders	44:0:0:6	M = 13.3, SD = 2.3	Stepwise Regression, correlation	Symptoms: tic disorder symptoms, ADHD symptoms*, behavioural problems*, obsessive–compulsive symptoms*, internalizing problems*, externalizing problems, anxiety*, depression*	low
Edvinsson (2018)	Sweden	EQ5D and EQ-VAS	Self	124	ADHD	63:61:0:0	M = 35.0, SD = 9.0	Mann–Whitney test	Symptoms: remission/ symptom reduction*	low
Engel-Yeger, 2022	Israel	WHOQOL-BREF	Self	46	ADHD	0:46:0:0	M = 27.6, SD = 9.2	Correlation	Symptoms: anxiety*, depression*	low
Evans (2020)	AUS	PedsQL	Parent	166	ADHD	166:0:0:0	M = 10.2, SD = 1.9	Correlation	Symptoms: autism symptoms/traits*, ADHD symptoms*, internalizing problems*, externalizing problems*, medication, Individual: age, Environmental: SES, parental mental health*	med
Flor (2017)	US	PedsQL	Parent	1347	autism	1024:204:0:119	Range = 2–17	t-test	Biology/physiology: complexity of autism (microcephaly and/or dysmorphology)*	low
Folostina (2023)	Greece, Romania	KINDL	Parent	125	autism	100:25:0:0	Range = 3–17	Correlation, Chi-square test, multiple linear regression	Individual: age, weight*, physical activity*, Environmental: parent age*, parent physical activity*	low
Galloway (2019)	Scotland	KIDSCREEN	Both	45	ADHD	40:5:0:0	M = 11.1	t-test, inter-correlation, multiple regression	Symptoms: autism symptoms/traits, ADHD symptoms*, learning disability symptoms, psychological comorbidities, Environmental: parent intervention*, parental mental health*	low
Georgiadou (2022)	Greece	Student with Disability Quality of Life Questionnaire and the adapted Satisfaction with Life Scale	Self	131	ID	70:61:0:0	M = 21.0, SD = 4.3	Correlation	Environmental: quality of schooling and services*	low
Gerlach (2021)	US; CAN	OASES	Self	505	stutter	290:210:5:0	M = 37.1, SD = 15.0	Hierarchical linear regression, correlation	Symptoms: stutter symptoms*, neuroticism*, Individual: age*, gender/sex*, sexuality, race/ethnicity*, stigma identity*, Environmental: SES*, self-help support group/self-help organizations*	low
Gortz-Dorten (2011)	Germany	KINDL	Both	589	ADHD	Not reported	Range = 6–17	Pearson's correlations	Symptoms: satisfaction with medication*	low
Grenwald-Mayes (2002)	US	QoL-Q	Self	37	ADHD	18:19:0:0	M = 24.3	Regression	Environmental: family functioning*	low
He (2019)	US	Q-LES-Q-S	Self	206	ADHD	105:0:0:101	M = 36.3, SD = 10.8	Linear regression (Higher levels of Self-Directedness)*	Individual: self-directedness*	low
Hematian (2009)	Iran	QoL-Q	Self	41	ID	24:17:0:0	M = 18.3	Stepwise regression	Individual: age, gender/sex, Environmental: SES*	low
Hesapcioglu (2014)	Turkey	PedsQL	Both	57	Tic disorders	43:14:0:0	Range = 6–16	Correlation	Symptoms: obsessive–compulsive symptoms, anxiety*, depression, Individual: positive self-perception*	low
Isaacs (2021)	US	GTS-QOL	Self	52	Tic disorders	35:17:0:0	M = 33	Spearman rank correlation	Symptoms: tic disorders symptoms*, ADHD symptoms*, obsessive–compulsive symptoms*, anxiety*, depression*	low
Jahan (2015)	Bangladesh	PedsQL	Parent	149	autism	115:34:0:0	M = 7.8, SD = 3.1	Student‚ t-test and ANOVA, correlation, linear regression	Symptoms: autism symptoms/traits, verbal communication*, medication, Individual: age, gender/sex, IQ*, vaccination, Environmental: parental age at pregnancy, SES*, age of first symptoms, age of diagnosis, parents’ consanguineous marriage, sibling with NDD, family structure	low
Karande (2012)	India	DCGM-37-S	Self	150	LD	121:29:0:0	M = 2.5, SD = 2.2	Effect sizes, multivariate logistic regression	Symptoms: ADHD symptoms, other unspecified problems, Functioning: academic problems, Individual: age, gender/sex*, Environmental: SES, sibling with NDD, family structure	low
Karci (2018)	Turkey	PedsQL	Both	50	ADHD	32:18:0:0	M = 14.5, SD = 1.7	Man-Whitney U test	Individual: gender/sex*	low
Kim, (2019)	Korea	PedsQL	Self	68	ADHD	68:0:0:0	M = 18.6, SD = 1.6	Pearson correlation, multiple regression	Symptoms: ADHD symptoms*, social problems*, thought problems/rule-breaking/aggression, oppositionality/ODD, conduct*, somatic problems*, affective problems*, internalizing problems*, externalizing problems*, anxiety*, depression*	low
Klang (2022)	Sweden	BBQ, EQ5S	Self	110	autism	35:70:0:5	M = 32.6, SD = 9.6	Correlation, multiple Linear regression	Symptoms: schizotypal personality*, depression, Individual: age, gender/sex*	low
Koedoot (2011)	Netherlands	HUI-3, EQ5D, EQ-VAS	Self	91	stutter	63:28:0:0	M = 36.0, SD = 14.7	t-test, correlation, multiple regression	Symptoms: stutter symptoms*, Individual: coping strategy*, Environmental: SES	low
Kuhlthau (2018)	US	PedsQL	Parent	4910	autism	4115:0:0:795	M = 6.2, SD = 3.5	Univariate regression, multivariate regression	Biology/physiology: physical health/well-being*, Symptoms: autism symptoms/traits*, obsessive–compulsive symptoms*, internalizing problems*, externalizing problems*, anxiety*, depression, bipolar*, gastrointestinal challenges*, seizures*, Individual: age*, gender/sex*, race/ethnicity*, IQ, healthy sleep*, Environmental: SES*, access to health care	low
Lachapelle (2005)	US; CAN; France and Belgium	QOL-Q	Self	182	ID	92:90:0:0		Discriminant function analysis	Individual: self-determination*	low
Lee (2020)	Korea	KIDSCREEN	Self	56	Tic disorders	47:9:0:0	M = 11.9, SD = 3.9	Correlation	Symptoms: tic disorder symptoms, anxiety*, depression*, Environmental: Expressed emotion within family: Critical style of communication*, Expressed emotion within family: Over-involved communication style	low
Lee (2022)	Korea	PedsQL	Self	43	ADHD	34:9:0:0	M = 9.2, SD = 1.7	Correlation Multiple linear regression	Symptoms: ADHD symptoms, anxiety*, depression*	low
Liu (2023)	China	PedsQL	Parent	363	Tic disorders	291:72:0:0	Median = 7.6	Multivariate logistic regression	Symptoms: tic disorder symptoms, behavioural problems*, Individual: age*, Environmental: SES, parenting style*, family functioning*, family structure, parental involvement in care	low
Logrieco (2022)	Italy	PedsQL	Parent	243	autism	209:0:0:34	M = 7.0, SD = 3.3	Correlation, Ordinary Least Squares regression	Symptoms: autism symptoms/traits*, verbal communication Individual: physical activity*, Environmental: SES, social support*, access to health care, parent age, family functioning*	low
Lucey (2019)	US	OASES	Self	33	stutter	24:9:0:0	M = 24.8	Pearson and Spearman correlation	Symptoms: social problems, depression, Individual: temperament	low
Malow (2016)	US	PedsQL	Parent	1515	autism	1267:0:0:248	Range = 4–10	Group difference	Symptoms: medication*	med
Mazon (2019)	France	AuQuEI	Self	45	autism; ID	Not reported	M = 14.3, SD = 1.4	Multiple regression	Symptoms: executive functioning*, Individual: age, IQ	low
McGuire (2015)	US	PedsQL	Self	24	Tic disorder	18:0:0:6	M = 11.3, SD = 2.7	Correlation multiple regression	Symptoms: tic disorder symptoms*	low
Meral (2015)	Turkey	KIDSCREEN	Parent	379	autism	298:76:0:5	M = 9.6, SD = 4.4	Correlation, regression	Symptoms: behavioural problems*, feeding problems*, Environmental: parenting style*	low
Mulraney (2019)	AUS	PedsQL	Parent	392	ADHD	335:0:0:57	M = 10.2, SD = 1.9	Correlation	Symptoms: ADHD symptoms*	low
Nicholson (2019)	Ireland	QoL Scale (self-report)	Self	82	ID	37:45:0:0	M = 35.7, SD = 10.3	ANOVA	Environmental: respite care	low
Ozboke (2021)	Turkey	PedsQL	Parent	31	autism	28:3:0:0	Range = 13–18	*t*-test, multiple regression	Symptoms: autism symptoms/traits*, motor skills, Functioning: adaptive functioning*	low
Park (2019)	Korea	PedsQL	Self	66	ADHD	55:11:0:0	M = 10.7, SD = 2.6	Correlation, regression	Symptoms: ADHD symptoms*, anxiety*, depression*	low
Payakachat (2014)	US	HUI	Parent	224	autism	194:30:0:0	M = 8.4, SD = 3.5	Correlation, Ordinary least squares regression	Symptoms: autism symptoms/traits*, behavioural problems*, internalizing problems*, externalizing problems, Functioning: adaptive functioning*, Individual: age, IQ*	low
Pearlman-Avnion (2017)	Israel	QoL-Q		31	autism	18:11:0:2	M = 27.8, SD = 11.3	*t*-test, correlation	Individual: sexual well-being, Environmental: social supports	low
Ragab (2020)	Egypt	PedsQL	Self	200	ADHD	123:77:0:0	Median = 9.0 years	Association, univariate regression	Symptoms: ADHD symptoms*, Individual: age, gender/sex*, Environmental: SES, geography*, parents’ age, parents’ marital status, sex/gender of parent informant*	low
Randall (2023)	US	ComQoL-I5	Self	27	ID	13:13:1:0	M = 45.1	Kruskal–Wallis test	Individual: employment status	low
Renty (2006)	Belgium	QOL.Q	Self	58	autism	43:0:0:15	M = 28.3, SD = 9.8	Pearson correlation	Symptoms: autism symptoms/traits, support received, Individual: IQ, Environmental: social support*, unmet support needs*	med
Rimmerman (2005)	Israel	QOL.Q	Self	127	ADHD	61:66:0:0	M = 28.7, SD = 4.7, M = 28.1, SD = 5.0	Correlation, regression	Symptoms: ADHD symptoms*, Functioning: medical disability*, Individual: age, Environmental: SES*, leisure activities in the community, social support*, living in an out of home programme	low
Rimmerman (2007)	Israel	QOL.Q	Self	127	ADHD	61:66:0:0	M = 28.4, SD = 4.8	Correlation, regression	Symptoms: ADHD symptoms*, Functioning: medical disability, Individual: age, Environmental: SES*, leisure activities in the community*, social support*, education setting*, living in an out of home programme	low
Roestorf (2022)	UK	WHOQOL-BREF, Personal well-being index, adult	Self	68	autism	0:17:0:51	M = 44.1, SD = 15.5	*t*-test, regression	Symptoms: autism symptoms/traits*, anxiety*, depression*, Individual: age*	low
Sahan (2020)	Turkey	PedsQL	Both	66	ADHD	66:0:0:0	Range = 6–10	Regression	Symptoms: ADHD symptoms*, specific learning disorder*, thought problems/rule-breaking/aggression, oppositionality/ODD, conduct*, anxiety*, fine motor skills*	low
Sasinthar (2022)	India	PedsQL	Parent	350	ID	Not reported	M = 12.6, SD = 3.8	Multilinear regression, Mann–Whitney U test and Kruskal–Wallis test	Symptoms: ID symptoms*, Individual: age, Environmental: SES, geography, parents’ consanguineous marriage*, parenting style	low
Sorkhi (2022)	Iran	WHOQOL-DIS-ID	Self	118	ID	70:48:0:0	M = 22.9, SD = 7.7	Regression	Individual: physical activity*, Environmental: leisure activity in the community*, social support*, access to health care*, parents’ marital status, parental mental health*	low
Stoeckel (2022)	Serbia	QOL.Q	Self	71	ID	39:32:0:0	Range = 29–67	MANOVA	Environmental: supportive housing	low
Torrente (2014)	Argentina	AAQoL	Self	35	ADHD	20:15:0:0	M = 31.2, SD = 9.5	Pearson correlation, regression	Symptoms: ADHD symptoms*, anxiety*, depression*, Individual: coping strategy*	low
Ueda (2021)	Japan	KINDL	Self	86	Tic disorders; autism; ADHD; LD; other DSM-5 NDD	70:16:0:0	M = 11.7, SD = 2.2	*t*-test, regression	Symptoms: depression*, Individual: healthy sleep*	low
VanAsselt-Goverts (2015)	Netherlands	IDQOL-16	Self	33	ID	16:17:0:0	M = 28.9	Pearson correlation	Environmental: Face to face contact of social network*, affection of social network*, preference of social network*, practical/informational support of social network*, structural characteristics of social network, connection (liking the same things as social network)	med
vanderKolk (2014)	Netherlands	KIDSCREEN	Parent	618	ADHD	509:109:0:0	M = 11.8	Multiple regression	Symptoms: psychological comorbidities*, response to medication*, Individual: age*, Environmental: SES*, parents’ marital status*, sibling with NDD*	med
Vincent (2020)	France	WHOQOL-BREF	Self	24	Asperger's syndrome	17:7:0:0	M = 22.2, SD = 3.4	Cross- analysis	Symptoms: ADHD symptoms, obsessive–compulsive symptoms, anxiety*, depression, Individual: gender/sex, Environmental: SES, social assistance, receiving care	high
White (2018)	CAN	QoL-Q	Self	30	autism	20:10:0:0	M = 21.3, SD = 3.3	Correlation	Individual: IQ, self-determination*	low
Wong (2019)	AUS	PedsQL	Self	63	ADHD	(50:13)	M = 14.28 SD = 2.07	Correlation, hierarchical regression	Symptoms: ADHD symptoms*, perceived effectiveness of medication, perceived effectiveness of behaviour therapy*, adherence to medication/therapy, General health perception: concern about illness*, beliefs/perception about cause*, perceived duration of diagnosis, symptoms/illness identity*, Individual: age. gender/sex, personal control over symptoms*, coping strategy*, sense of coherence/understanding*	low
Yarar (2022)	UK	WHOQOL-BREF	Self	79	autism	(61:18)	M = 44.96 years, SD = 15.36	ANOVA, correlation	Symptoms: autism symptoms/traits*, obsessive–compulsive symptoms*, anxiety*, depression*, Individual: age, IQ	low
Zinner (2012)	US	PedsQL	Both	206	Tic disorders	Group 1 (40:15) Group 2 (129; 22)	M = 12.2, SD = 2.2	t-test	Environmental: experiencing peer victimization*	low

Informant is reported as parent, self, or both. Sex/gender is reported as (male:female:Non-binary/agender:other/not-specified). Age is reported in years (M: mean, SD: standard deviation) unless otherwise stated

*ADD* attention deficit disorder, *AAQoL*adult ADHD Quality Of Life scale, *ADOS* autism diagnostic observation schedule, *ADOS* autism diagnostic observation schedule, *AuQuEI* autoquestionnaire qualité de vie enfant imagé, *ASQoL* autism-specific quality of life, *BBQ* Brunnsviken Brief Quality of Life Scale, *CBCL* child behavior checklist, *CCQOLI* cross-cultural quality of life indicators, *ComQoL-I5* Comprehensive Quality of Life Scale Intellectual/Cognitive Disability 5th Edition, *CPRS* Conners' Parent Rating Scales, *EQ5D* EuroQol 5-dimensions, *EQ-VAS* EuroQol Visual Analog Scale, *GTS-QOL* Gilles de la Tourette Syndrome-Quality of Life Scale, *HQLS* Heinrichs Quality of Life Scale, *HRQOL* health-related quality of life, *ID* intellectual disability, *IDQOL* intellectual disability quality of life, *LD* learning disabilities, *MASC* Multidimensional Anxiety Scale for Children, *NDD* neurodevelopmental disorders, *OASES* overall Assessment of the Speaker's experience of stuttering, *OCD* obsessive–compulsive disorder, *OLS* Orientation to Life Scale, *PDD/NOS* pervasive developmental disorder/not otherwise specified, *PedsQL* pediatric quality of life inventory, *QoL* quality of life, *QoL-Q* quality of life questionnaire, *Q-LES-Q-S* quality of life enjoyment and satisfaction questionnaire, *SCQ* social communication questionnaire, *SES* socioeconomic status, *SLD* specific learning disorder, *SVE-ServQual %SM* Service Quality Scale, *SPQ-BR-32* social phobia questionnaire, *HUI* The Health Utilities Index, *TNO-AZL* children's quality of life, *WHOQOL-BREF* world health organization quality of life—BREF, *WHOQOL-DIS-ID* world health organization quality of life—disability module—intellectual disability, *WM* working memory, *WOCS* ways of coping, *YQOL-R* youth quality of life instrument-research

*indicates significance

### Study Populations

The most frequently studied diagnoses were autism (*n* = 23) and ADHD (*n* = 22), followed by intellectual disorder (*n* = 14), tic disorders (*n* = 11), and stutter (*n* = 5). The number of studies investigating pediatric (< 21 years), and adult groups were 37 and 39, respectively, with one study examining both groups. Of the reviewed studies, only two reported HRQoL predictors across multiple diagnosis categories. This included one study on tic disorders, autism, ADHD, and learning disorder, and another on autism and intellectual disability.

For the studies that reported sex and/or gender (total participants 16,639), there were 3924 female (24%), 12,685 male (76%), 6 non-binary (< 1%), and 24 not-specified/other (< 1%) participants. Twenty-one studies reported socioeconomic status indicators (composite scores, income, employment, or education).

### HRQoL Measurement

Across the reviewed studies, the most frequently used instrument used to assess HRQoL was the Pediatric Quality of life inventory (Varni et al., 2001) (PedsQL; *n* = 25), followed by the Quality of Life Questionnaire (QoL-Q; *n* = 11). Beyond these, the measures used in the reviewed literature were highly heterogeneous.

### Analytical Approaches

To quantify the association between HRQoL and predictors, a wide variety of methodological approaches were employed in the reviewed studies. These included computation of correlation coefficients, comparisons of groups defined on predictor variables (e.g. analysis of variance, t tests), and regression analysis.

### Predictors of HRQoL

With reference to the Revised Wilson and Cleary model, the most frequently studied predictors of HRQoL were in domains of symptoms and individual factors. Significant gaps were evident in studies examining predictors in domains of biology/physiology, functioning, environment, and general health perceptions, within and across conditions, as described below.

#### Biology/Physiology (Table 2)

**Table 2:** Biology/physiology predictors of quality of life

Biology/physiology	Autism	ADHD	ID
Physical health/well-being	+ Caron et al. (2022); Kuhlthau et al. (2018) 0 Caron et al. (2022)		0 Cramm & Nieboer (2012)
Microcephaly and/or dysmorphology	**−** Flor et al. (2017)		

+ = Positive association

− = Negative association

0 = Not significant

Four of the 78 studies reported on predictors related to this domain (3 autism; 1 ID), with a focus on physical health/wellbeing (e.g. physical health conditions, sensory disorders, chronic pain, migraines or headaches), and microcephaly and dysmorphology. These studies revealed positive or null associations between physical health variables and HRQoL.

#### Symptoms (Table 3)

**Table 3:** Symptom predictors of quality of life

Symptoms	Autism	ADHD	ID	Stutter	LD	TD	Cross-Diagnosis
*Core domains*							
Autism symptoms/traits	**− **Caron et al. (2022); Corbera et al. (2021); de Vries et al. (2018); Kuhlthau et al. (2018); Logrieco et al. (2022); Ozboke et al. (2021); Payakachat et al. (2014); Roestorf et al. (2022); Yarar et al. (2022) 0 Adams et al. (2019); Caron et al. (2022); Corbera et al. (2021); Jahan et al. (2015); Kuhlthau et al. (2018); Payakachat et al. (2014); Renty & Roeyers (2006); Yarar et al. (2022)	**−** (Ahnemark et al. (2018); Evans et al. (2020) 0 Ahnemark et al. (2018); Galloway et al. (2019)					
Stutter symptoms				**−** Boyle (2015); Gerlach et al. (2021); Koedoot et al. (2011) 0 Carter et al. (2017); Gerlach et al. (2021); Koedoot et al. (2011)			
ID symptoms			**−** Sasinthar et al. (2022)				
Tic disorder symptoms						**−** Cavanna et al. (2012); Eapen et al. (2016); Isaacs et al. (2021); McGuire et al. (2015) 0 Bernard et al. (2009); Cavanna et al. (2012); Eapen et al. (2016); Eddy et al. (2011); Isaacs et al. (2021); H. Lee et al. (2020); Liu et al. (2023); McGuire et al. (2015)	
ADHD symptoms	0 Caron et al. (2022); Vincent et al. (2020)	**−** Ben-Dor Cohen et al. (2021); Evans et al. (2020); Galloway et al. (2019); Kim (2019); Mulraney et al. (2019); Park et al. (2019); Ragab et al. (2020); Rimmerman et al. (2005), (2007); Sahan et al. (2020); Torrente et al. (2014); Wong et al. (2019) 0 Ahnemark et al. (2018); Evans et al. (2020); Galloway et al. (2019); Lee et al. (2022); Mulraney et al. (2019); Park et al. (2019); Rimmerman et al. (2005); Wong et al. (2019)			0 Karande & Venkataraman (2012)	**−** Bernard et al. (2009); Eapen et al. (2016); Eddy et al. (2011); Isaacs et al. (2021) 0 Cavanna et al. (2012); Eapen et al. (2016)	
Years since diagnosis						0 Bernard et al. (2009)	
Remission/ symptom reduction		** + ** Edvinsson & Ekselius (2018)					
Learning disability symptoms	0 Caron et al. (2022)	0 Galloway et al. (2019)					
Specific learning disorder (SLD)		**−** Sahan et al. (2020)					
Verbal communication	** + ** Jahan et al. (2015) 0 Logrieco et al. (2022)						
Executive functioning	** + ** Dijkhuis et al. (2017); Mazon et al. (2019) 0 de Vries et al. (2018)						
Sensory disorder	**−** Caron et al. (2022) 0 Caron et al. (2022)						
*Mental/behavioural*							
Behavioural problems	**−** Meral & Fidan (2015); Payakachat et al. (2014) 0 Payakachat et al. (2014)		0 Balboni et al. (2020)			**−** Eddy et al. (2011); Liu et al. (2023)	
Obsessive–compulsive symptoms	**−** Kuhlthau et al. (2018); Yarar et al. (2022) 0 Vincent et al. (2020)					**−** Bernard et al. (2009); Eddy et al. (2011); Isaacs et al. (2021) 0 Cavanna et al. (2012); Hesapçıoğlu et al. (2014)	
Neuroticism				**−** Gerlach et al. (2021)			
Social problems		**−** Kim (2019)		0 Lucey et al. (2019)			
Thought problems/rule-breaking/aggression, oppositionality/ODD, conduct		**−** Kim (2019); Sahan et al. (2020)					
Somatic problems		0 Kim (2019) **−** Kim (2019)					
Affective problems		**−** Kim (2019)					
Emotional dysregulation		**−** Ben-Dor Cohen et al. (2021)					
Internalizing problems	**−** Kuhlthau et al. (2018); Payakachat et al. (2014)	**−** Evans et al. (2020); Kim (2019)				**−** Eddy et al. (2011)	
Externalizing problems	**−** Kuhlthau et al. (2018) 0 Payakachat et al. (2014)	**−** Evans et al. (2020); Kim (2019)				0 Eddy et al. (2011)	
Schizotypal personality	**−** Klang et al. (2022) 0 Klang et al. (2022)						
Anxiety	− Adams et al. (2019), (2020); Caron et al. (2022); Kuhlthau et al. (2018); Roestorf et al. (2022); Vincent et al. (2020); Yarar et al. (2022) 0 Adams et al. (2019)	**−** Ahnemark et al. (2018); Engel-Yeger (2022); Kim (2019); Lee et al. (2022); Park et al. (2019); Sahan et al. (2020); Torrente et al. (2014) 0 Ahnemark et al. (2018); Crawford et al. (2015); Park et al. (2019)				**−** Eddy et al. (2011); Hesapçıoğlu et al. (2014); Isaacs et al. (2021); Lee et al. (2020) 0 Eddy et al. (2011); Hesapçıoğlu et al. (2014)	
Depression	**−** Roestorf et al. (2022); Yarar et al. (2022) 0 Klang et al. (2022); Kuhlthau et al. (2018); Vincent et al. (2020)	**−** Ahnemark et al. (2018); Engel-Yeger (2022); Kim (2019); Lee et al. (2022); Park et al. (2019); Torrente et al. (2014) 0 Ahnemark et al. (2018); Torrente et al. (2014)	**−** Cramm & Nieboer, 2012)	0 Lucey et al. (2019)		**−** Eddy et al. (2011); Isaacs et al. (2021); H. Lee et al. (2020) 0 Hesapçıoğlu et al. (2014)	**−** Ueda et al. (2021)
Mood disorders	**−** Caron et al. (2022)						
Bipolar	**−** Kuhlthau et al. (2018)						
Psychological comorbidities		**−** Ahnemark et al. (2018); van der Kolk et al. (2014) 0 Galloway et al. (2019)				**−** Eapen et al. (2016)	
Other unspecified problems					0 Karande & Venkataraman (2012)		
Self-injurious behaviour						**−** Cavanna et al. (2012)	
*Physical health*							
Gastrointestinal challenges	**−** Kuhlthau et al. (2018)						
Feeding problems	**−** Meral & Fidan (2015)						
Fine motor skills		** + ** Sahan et al. (2020)					
Motor skills	0 Ozboke et al. (2021)						
Seizures	**−** Capal et al. (2020); Kuhlthau et al. (2018)						
*Interventions*							
Medication	**−** Malow et al. (2016) 0 Caron et al. 2022; Jahan et al. (2015)	0 Ben-Dor Cohen et al. (2021); Evans et al. (2020)					
Satisfaction with medication		** + ** Gortz-Dorten et al. (2011)					
Response to medication		** + ** van der Kolk et al. (2014)					
Perceived effectiveness of medication		0 Wong et al. (2019)					
Behaviour therapy (perceived effectiveness)		** + ** Wong et al. (2019) 0 Wong et al. (2019)					
Adherence to medication/therapy		0 Wong et al. (2019)					
Support received	0 Renty & Roeyers (2006)						

+ = Positive association

− = Negative association

0 = Not significant

This domain was the most frequently studied predictor of HRQoL (autism: 20, ADHD: 19, ID: 3, tic disorder: 9, stutter: 5, learning disorder: 1, cross-diagnosis: 1). We grouped the symptoms investigated into four categories: (1) symptoms/features associated with core domains of each neurodevelopmental condition, (2) mental health/behavioural features, (3) physical symptoms, and (4) interventions aimed at reducing symptom intensity/impact. The existing literature on core domains was heavily focused on features associated with autism (15 studies) and ADHD (22 studies). Cross-diagnosis studies of symptoms were scarce and limited to investigation of ADHD symptoms (autism: 2 studies, learning disorders: 1 study, tic disorders: 5 studies) and autism features (ADHD: 3 studies). Overall, several studies reported a negative association between symptom intensity in the core domains and HRQoL across diagnoses (*n* = 21), although null findings were common (*n* = 32).

In terms of mental health/behaviour, the impact of mental health symptoms on HRQoL was most frequently investigated, with a significant focus on anxiety (19 studies) and depression (18 studies). These symptoms were overwhelmingly associated with decreased HRQoL across diagnoses (31 studies), with a small number of studies reporting null findings (13 studies). Studies examining the impact of interventions on HRQoL mainly included participants with ADHD (5 studies), followed by autism (4 studies). This very small body of literature showed a differential impact of interventions in ADHD and autism, with very preliminary suggestion of potentially positive impact in ADHD, and null or negative findings in autism. Studies of physical health were relatively limited and restricted to autism and ADHD.

#### Functioning (Table 4)

**Table 4:** Functioning predictors of quality of life

Functioning Predictors	Autism	ADHD	ID	LD
Adaptive functioning	+ Ozboke et al. (2021; Payakachat et al. (2014)		** + ** Balboni et al. (2020)	
Medical disability/% medical disability		− Rimmerman et al. (2005) 0 Rimmerman et al. (2005, 2007)		
Activities of daily living			+ Chou et al. (2007) 0 Chou et al. (2007); Cramm & Nieboer (2012)	
Academic problems				0 Karande & Venkataraman (2012)

+ = Positive association

− = Negative association

0 = Not significant

The literature on predictors of HRQoL related to functioning was very sparse and included investigations of daily living skills and performance of everyday activities (autism: 2, ADHD: 2, ID: 3, LD:1). The majority of the reviewed studies suggested a positive association between adaptive functioning skills and HRQoL across neurodevelopmental conditions.

#### General Health Perceptions (Table 5)

**Table 5:** General health perception predictors of quality of life

General health perceptions	ADHD	Stutter
Concern about illness	**−** Wong et al. (2019)	
Beliefs/perception about cause	− Wong et al. (2019) 0 Wong et al. (2019)	
Perceived duration of diagnosis	0 Wong et al. (2019)	
Symptoms/illness identity	− Wong et al. (2019)	** + ** Boyle (2015)

+ = Positive association

− = Negative association

0 = Not significant

There was very limited investigation of the impact of health perceptions on HRQoL across neurodevelopmental conditions (ADHD: 1, stutter: 1). The studied predictors included concerns about illness/condition, beliefs and perceptions about cause, perceived duration of symptoms, and identity.

#### Individual Characteristics (Table 6)

**Table 6:** Individual predictors of quality of life

Individual	Autism	ADHD	ID	Stutter	LD	TD	Cross-Diagnosis
*Demographics*							
Age	** + ** Roestorf et al. (2022) **−** Caron et al. (2022); Dijkhuis et al. (2017); Kuhlthau et al. (2018) 0 Adams et al. (2019); Caron et al. (2022); Folostina et al. (2023); Jahan et al. (2015); Klang et al. (2022); Mazon et al. (2019); Payakachat et al. (2014); Yarar et al. (2022)	**−** van der Kolk et al. (2014) 0 Ahnemark et al. (2018); Evans et al. (2020); Ragab et al. (2020); Rimmerman et al. (2005), (2007); Wong et al. (2019)	**−** Chou et al. (2007) 0 Balboni et al. (2020); Crawford et al. (2015); Hematian et al. (2009); Sasinthar et al. (2022)	** + ** Boyle (2015) **−** Carter et al. (2017); Gerlach et al. (2021) 0 Gerlach et al. (2021)	0 Karande & Venkataraman (2012)	** + ** Liu et al. (2023) 0 Bernard et al. (2009)	
Gender/sex (Male)	** + ** Caron et al. (2022); Karci et al. (2018); Kuhlthau et al. (2018) 0 Dijkhuis et al. (2017); Jahan et al. (2015); Klang et al. (2022); Vincent et al. (2020) **−** Klang et al. (2022)	** + ** Ahnemark et al. (2018); Ragab et al. (2020) 0 Wong et al. (2019)	0 Balboni et al. (2020); Chou et al. (2007); Hematian et al. (2009)	** + ** Gerlach et al. (2021) 0 Boyle (2015); Gerlach et al. (2021)	** + ** Karande & Venkataraman (2012)		
Sexuality (Heterosexual)				0 Gerlach et al. (2021)			
Minority race/ethnicity	** + ** Kuhlthau et al. (2018) 0 Caron et al. (2022)			** + ** Gerlach et al. (2021)			
Employment status (full time)	** + ** Caron et al. (2022)	** + ** Ahnemark et al. (2018)	** + ** Chou et al. (2007) 0 Balboni et al. (2020); Chou et al. (2007); Randall et al. (2023)				
*Anthropomorphic*							
Weight	** + ** Folostina et al. (2023) 0 Folostina et al. (2023)						
*Psychological characteristics*							
IQ	** + ** Corbera et al. (2021); Jahan et al. (2015); Payakachat et al. (2014) 0 de Vries et al. (2018); Kuhlthau et al. (2018); Mazon et al. (2019); Payakachat et al. (2014); Renty & Roeyers (2006); White et al. (2018); Yarar et al. (2022)	0 Ahnemark et al. (2018)	0 Crawford et al. (2015)				
Temperament				0 Lucey et al. (2019)			
Personal control over symptoms		** + ** Wong et al. (2019) 0 Wong et al. (2019)					
Coping strategy		** + ** Torrente et al. (2014); Wong et al. (2019) 0 Wong et al. (2019)		** + ** Koedoot et al. (2011) 0 Koedoot et al. (2011)			
Stigma identity (salience, centrality, concealment, verbal self-disclosure)				** + ** Gerlach et al. (2021) 0 Gerlach et al. (2021)			
Self-determination	** + ** White et al. (2018)		** + ** Lachapelle et al. (2005)				
Sense of coherence/understanding		** + ** Wong et al. (2019) 0 Wong et al. (2019)					
Feeling of freedom from worries/feeling bad/peer rejection		** + ** Dolgun et al. (2014)					
Positive self-evaluation in academics		** + ** Dolgun et al. (2014) 0 Dolgun et al. (2014)					
Self directedness		** + ** He et al. (2019)					
Reward sensitivity	**−** de Vries et al. (2018)						
Emotion processing	0 Dijkhuis et al. (2017)						
Self-efficacy for verbal communication				**−** Carter et al. (2017)			
Sexual well-being	0 Pearlman-Avnion et al. (2017)						
Empowerment				** + **Boyle (2015)			
Involvement in treatment				** + ** Boyle (2015)			
Positive self-perception		** + ** Dolgun et al. (2014)	** + ** Albuquerque (2012)	** + ** Boyle (2015)		** + ** Hesapçıoğlu et al. (2014) 0 Hesapçıoğlu et al. (2014)	
*Healthfulness behaviours*							
Healthy sleep	** + ** Kuhlthau et al. (2018)						** + ** Ueda et al. (2021)
Vaccination	0 Jahan et al. (2015)						
Physical activity	** + ** Folostina et al. (2023); Logrieco et al. (2022) 0 Folostina et al. (2023)		** + ** Sorkhi et al. (2022) 0 Sorkhi et al. (2022)			** + ** Doja et al. (2018)	

+ = Positive association

− = Negative association

0 = Not significant

Forty-eight studies investigated the variables related to individual characteristics (autism: 19, ADHD: 10, stutter: 5, LD: 1, TD: 4, ID: 9). We grouped the variables investigated as predictors into four categories: (1) demographics, (2) psychological factors, (3) anthropomorphic, and (4) healthfulness behaviours. Demographics variables were most frequently investigated across diagnoses, with a focus on age (autism: 11, ADHD: 7, stutter: 3, LD: 1, TD: 2, ID: 5), sex/gender (autism: 7, ADHD: 3, stutter: 3, LD: 1, TD: 2, ID: 3), and employment (autism: 1, ADHD: 1, ID: 3). The effects of age on HRQoL were mixed, whereas male gender and employment were most frequently associated with increased HRQoL. Beyond age and sex/gender, there was a paucity of studies examining the effects of demographics such as race/ethnicity and sexual orientation.

In terms of psychological factors, IQ was most frequently studied as an individual factor; however, these studies were mainly limited to autism (9 studies), with 1 study related to ADHD and 1 study focused on intellectual disability. Of these, seven studies reported null associations between IQ and HRQoL, consistent with the findings in ADHD (1 study) and ID (1 study). Positive self-perception was also studied in four publications, with reports of positive association with HRQoL. For healthfulness behaviours, physical activity was examined in 4 studies (autism: 2, ID: 1, TD: 1), with all studies reporting either a positive or null association between physical activity and HRQoL.

#### Environmental Characteristics (Table 7)

**Table 7:** Environmental predictors of quality of life

Environmental	Autism	ADHD	ID	Stutter	LD	TD
*Prenatal/birth factors*						
Parental age at pregnancy	0 Jahan et al. (2015)					
Family history						**−** Cavanna et al. (2012)
*Social environment*						
SES	** + ** Caron et al. (2022); Jahan et al. (2015); Kuhlthau et al. (2018) **−** Kuhlthau et al. (2018) 0 Caron et al. (2022); Jahan et al. (2015); Kuhlthau et al. (2018); Logrieco et al. (2022); Vincent et al. (2020)	** + ** Ahnemark et al. (2018); Rimmerman et al. (2005), (2007); van der Kolk et al. (2014) 0 Evans et al. (2020); Ragab et al. (2020); Rimmerman et al. (2005)	** + ** Chou et al. (2007); Hematian et al. (2009) 0 Chou et al. (2007); Cramm & Nieboer (2012); Sasinthar et al. (2022)	**−** Gerlach et al. (2021) 0 Gerlach et al. (2021); Koedoot et al. (2011)	0 Karande & Venkataraman (2012)	0 Liu et al. (2023)
Social assistance	0 Vincent et al. (2020)					
Violence history	**−** Caron et al. (2022)					
Leisure activities in the community		** + ** Rimmerman et al. (2007) 0 Rimmerman et al. (2005, 2007)	** + ** Sorkhi et al. (2022)			
Experiencing peer victimization						**−** Zinner et al. (2012)
*Social supports*						
Social support	** + ** Logrieco et al. (2022); Renty & Roeyers (2006) 0 Pearlman-Avnion et al. (2017)	** + ** Rimmerman et al. (2005, 2007) 0 Rimmerman et al. (2005, 2007)	** + ** Crawford et al. (2015); Sorkhi et al. (2022) 0 Cramm & Nieboer (2012)	** + ** Boyle (2015)		
Face to face contact of social network			** + ** van Asselt-Goverts et al. (2015)			
Affection of social network			** + ** van Asselt-Goverts et al. (2015)			
Preference of social network (preference for contact with the person, liking the contact)			** + ** van Asselt-Goverts et al. (2015)			
Practical/informational support of social network			** + ** van Asselt-Goverts et al. (2015)			
Structural characteristics of social network (size, telephone/internet frequency, length, accessibility)			0 van Asselt-Goverts et al. (2015)			
Connection (liking the same things as social network)			0 van Asselt-Goverts et al. (2015)			
*Resources*						
Education setting (inclusive versus special education)		** + ** Rimmerman et al. (2007) 0 Rimmerman et al. (2007)				
Quality of schooling and services			** + ** Georgiadou et al. (2022) 0 Georgiadou et al. (2022)			
Geography		**−** Ragab et al. (2020)	** + ** Chou et al. (2007) 0 Balboni et al. (2020); Chou et al. (2007); Sasinthar et al. (2022)			
Living in an out of home programme		0 Rimmerman et al. (2005, 2007)				
Supportive housing			** + ** Stoeckel et al. (2022)			
Access to health care	** + ** Kuhlthau et al. (2018) 0 Logrieco et al. (2022)		** + ** Sorkhi et al. (2022) 0 Sorkhi et al. (2022)			
Receiving care	0 Vincent et al. (2020)					
Parent intervention		**−** Galloway et al. (2019)				
Self-help support group/self-help organizations				** + ** Boyle (2015); Gerlach et al. (2021) 0 Boyle (2015)		
Speech training				0 Gerlach et al. (2021)		
Respite care			0 Nicholson et al. (2019)			
Clinician diagnostic confidence						**−** Cavanna et al. (2012)
Age of First Symptoms	**−** Jahan et al. (2015)					
Age of diagnosis	**−** Caron et al. (2022) 0 Caron et al. (2022); Jahan et al. (2015)					
Unmet support needs	**−** Renty & Roeyers (2006)					
*Family context*						
Parent age	** + ** Folostina et al. (2023) 0 Folostina et al. (2023); Logrieco et al. (2022)	0 Ragab et al. (2020)				
Parent’s consanguineous marriage	0 Jahan et al. (2015)		** + ** Sasinthar et al. (2022)			
Parents’ marital Status (married/living together)		**−** van der Kolk et al. (2014) 0 Ragab et al. (2020)	0 Sorkhi et al. (2022)			
Having at least one child		0 Ahnemark et al. (2018)				
Sibling with NDD	0 Jahan et al. (2015)	**−** van der Kolk et al. (2014)			0 Karande & Venkataraman (2012)	
Poor parental mental health		**−** Evans et al. (2020); Galloway et al. (2019) 0 Galloway et al. (2019)	**−** Cramm & Nieboer (2012); Sorkhi et al. (2022) 0 Cramm & Nieboer (2012); Sorkhi et al. (2022)			
Parenting style	** + ** Meral & Fidan (2015)		0 Sasinthar et al. (2022)			** + ** Liu et al. (2023)
Parent informant (Male)		** + ** Ragab et al. (2020)				
Parent physical activity	** + ** Folostina et al. (2023) 0 Folostina et al. (2023)					
Family functioning	** + ** Logrieco et al. (2022)	** + ** Grenwald-Mayes (2002)				** + ** Liu et al. (2023)
Family structure	0 Jahan et al. (2015)				0 Karande & Venkataraman (2012)	0 Liu et al. (2023)
Expressed emotion within family: Critical style of communication						**−** Lee et al. (2020)
Expressed emotion within family: Over-involved communication style						0 Lee et al. (2020)
Parental Involvement in Care						0 Liu et al. (2023)

+ = Positive association

− = Negative association

0 = Not significant

Our results revealed 36 studies which examined the association between HRQoL and environmental characteristics across diagnostic groups (autism: 9, ADHD: 8, stutter: 3, tic disorder: 4, LD: 1, ID: 11). The predictors examined in these studies were clustered into six categories: (1) prenatal/birth factors, (2) social environment, (3) social supports, (4) physical environment, (5) resources, and (6) family context. The literature on prenatal/birth factors was limited to two studies. Among predictors related to the social environment, socioeconomic status was most commonly investigated across diagnoses, with highly mixed findings reported within and across diagnoses (positive, null, and negative associations). The impact of social supports on HRQoL was also frequently examined across diagnoses (autism: 3, ADHD, 2, stutter: 1, ID, 4), with eight studies reporting positive and five studies reporting null associations. Predictors related to resources included healthcare resources, and academic and physical environments. Across diagnoses, these resources were associated with positive impact on HRQoL across the majority of studies. Finally, variables related to family context were investigated in 16 studies, with the majority suggesting an association between positive family context (e.g. parental mental health, family function) and improved HRQoL.

## Discussion

We conducted this systematic review to synthesize the literature findings related to transdiagnostic predictors of HRQoL across neurodevelopmental conditions. Our review revealed less than 30 published studies for each condition meeting our review criteria. These studies mainly focus on autism and ADHD, with a significant paucity of literature on HRQoL predictors in communication disorder, language disorder, speech disorder, speech sound disorder, fluency disorder, motor disorder, developmental coordination disorder, or stereotypic movement disorder. This is a critical gap given the prioritization of quality of life as an outcome by clinicians (Lord et al., 2022) and the neurodivergent communities (Oakley et al., 2021).

Cross-diagnosis investigation of HRQoL predictors was highly limited in the literature, despite the fact that many of the examined variables transcend diagnostic boundaries. This is a significant gap as many symptoms overlap largely among neurodevelopmental conditions (Craig et al., 2016; Stern & Robertson & 1997, Hulsbosch et al., 2021; Nippold & Schwarz, 1990). Similarly, influencers related to adaptive functioning, health perceptions, and demographics, and environmental context can also be shared across individuals with neurodevelopmental conditions.

The results of this review provide very preliminary suggestions on potentially shared predictors of HRQoL across HRQoL. In particular, very early patterns were observed to suggest positive associations between HRQoL and adaptive functioning, male sex/gender, positive self-perception, physical activity, resources, and positive family context, and negative associations with core and mental health symptoms. It is important to note that although these predictors may also be relevant to HRQoL in neurotypical populations, neurodivergent populations may be more likely to experience negative predictors and at greater intensity (e.g. mental health). Reducing exposure to these factors through timely access to care and environmental adaptations and supports can therefore contribute to greater HRQoL.

The only domain where preliminary differential effects were observed across conditions was the impact of interventions. These results suggested a pattern of positive association in ADHD and null or negative findings in autism. Although very preliminary, these patterns are consistent with previous literature suggesting increases in QoL associated with medication use in ADHD (Agarwal et al., 2012; Coghill, 2010; Coghill et al., 2017) and mixed perceptions of interventions in autism (Schuck et al., 2022). These results must be interpreted with caution given that we did not carry out a meta-analysis to quantify effect sizes.

### Measurement and Analysis

The most frequently used instrument used for measuring HRQoL in the reviewed literature was the Pediatric Quality of Life Inventory (PedsQL). The PedsQL is a 23-item questionnaire investigating HRQoL across four domains of physical functioning, emotional functioning, school functioning, and social functioning (Varni et al., 2001). This measure includes both self- and parent-report versions, and age-appropriate versions for children 2–18 years old. In adult HRQoL studies, QoL-Q (Schalock & Keith, 1993) was most commonly used. This is a 40-item questionnaire with four subscales: personal life and satisfaction, competence and productivity, empowerment and independence, and social belonging and community integration. Overall, we found a heterogeneity of instruments used, which challenged the interpretation and compatibility of results across studies. It is also important to note that our understanding of the validity of existing HRQoL measures in neurodivergent communities is very limited as these measures are often not co-created or validated with neurodivergent individuals. This is critical as perceptions of HRQoL may differ between neurotypical and neurodivergent populations. For example, the subdomains related to social functioning may be valued differently by neurotypical and neurodivergent populations. These suggestions are similar to those in existing reviews critiquing HRQoL studies in neurodevelopmental populations, suggesting that an NDD-specific HRQoL instrument is needed (Evers et al., 2022). We are aware of one study (McConachie et al., 2018) which addresses these challenges by examining the psychometric properties of the WHOQoL-BREF in autistic adults and co-created nine additional autism-specific items. Additional studies to further understand HRQoL from the perspectives of other neurodivergent communities are an important area for future research.

In addition to differences in instruments used, a variety of analytical approaches were employed in the reviewed literature to quantify the associations between HRQoL and the hypothesized predictors. These methodological differences, including differences in assumptions of linearity and normality, and inclusion of covariates and interaction terms, may contribute to the heterogeneity of findings in this field.

### Predictors of HRQoL

HRQoL is a multi-dimensional construct and impacted by several interacting domains. To reflect this complexity, we grounded our analyses in a theoretical model of HRQoL, the Revised Wilson and Cleary model (Ferrans et al., 2005). With reference to this model, the most commonly investigated predictors of HRQoL were in the symptom domain. This included both studies examining core features of neurodevelopmental conditions as well as co-occurring symptoms. For the latter, our results suggest that mental health, and specifically anxiety and depression, may be transdiagnostic domains which negatively impact HRQoL in neurodevelopmental conditions. Given the high prevalence of these symptoms in neurodevelopmental conditions [e.g. In autism, 20 and 11% prevalence of anxiety and depressive disorder, respectively (Lai et al., 2019)], future research in this area, including a meta-analysis, is highly encouraged.

Physical health is also a key area for future research in neurodivergent children as there is a sizable body of evidence in community samples suggesting that physical health may positively impact HRQoL (Cordova et al., 2021; Davies et al., 2019; Gu et al., 2020; Redondo-Tebar et al., 2019; Schafer et al., 2016; Tsiros et al., 2017), but our review found very few studies on this topic.

In addition to symptoms, our review revealed that individual characteristics were also frequently studied as predictors of HRQoL across neurodevelopmental populations, with a significant focus on age and sex/gender. Despite a growing body of literature examining the impact of age on HRQoL, the findings were highly mixed. For sex/gender, our results suggest a potential association of male sex/gender with increased HRQoL across neurodevelopmental conditions. Future studies in this area are needed to better understand the nature of this association. Additionally, these findings must be interpreted in the context that the majority of studies did not differentiate between sex as a biological variable and gender as a social identity, and study samples did not include gender-diverse participants, with less than 1% of the sample across all studies having a non-binary gender identity. There was also a significant gap in understanding the impact of other demographic variables, such as race/ethnicity/Indigeneity, immigration status, and other dimensions of identity. These can impact well-being through access to health resources (Khanlou et al., 2017), intergenerational trauma (Czyzewski, 2011), and experiences of discrimination (Benner et al., 2018). In terms of other individual predictors, the literature reports were sparse, but a handful of studies suggested positive associations between HRQoL and positive self-perception and physical activity. Future studies are needed to further understand these associations.

In the domain of environmental predictors, our review found highly mixed findings with respect to SES. Our results highlighted social supports and family functioning as potential avenues for future investigation as preliminary positive associations with HRQoL were reported. At the same time, our results revealed gaps in understanding other environmental influencers, such as access to care and resources, accommodations, inclusion, and acceptance, social and environmental barriers, as well as other factors that impact the person-environment fit (Lord et al., 2022). Timely access to healthcare resources and social support also significantly impacts outcomes in neurodevelopmental conditions and likely predict HRQoL. These findings are in line with other reviews investigating predictors in single neurodevelopmental conditions (Chiang & Wineman, 2014; Sevastidis et al., 2023).

A significant literature gap was also found in the domain of functioning (ability to complete tasks of daily life). This is a key area for future studies of HRQoL in neurodevelopmental conditions as functioning may help to disentangle the distinction between individual differences and disability.

Lastly, most reviewed studies focused on predictors in single domains impacting HRQoL in isolation. This isolated study of HRQoL predictors does not reflect the multi-dimensional nature of HRQoL and the interconnectedness among the various influences. Given the complexity of the HRQoL construct, future studies should consider the interrelations among the various domains impacting HRQoL. Examples include examining the effect of sociodemographic and environmental variables as moderators of the associations among HRQoL, symptoms, and functioning. Grounding such investigating in a theoretical model can further contextualize the findings of future studies.

## Strengths and Limitations

This study had various strengths. The transdiagnostic approach of this study allows the exploration of HRQoL predictors that transcend diagnostic boundaries and reflects the large overlap among neurodevelopmental conditions. In addition, grounding our analyses in a theoretical model allowed us to explore HRQoL predictors with a global and multi-dimensional lens.

The findings of this review should be interpreted in the context of several limitations. We considered HRQoL as a single dimensional construct (total score). This choice was made due to the large heterogeneity in domains included in various HRQoL instruments, limiting the ability to capture subscales. Additionally, we did not consider interactions among HRQoL domains or their predictors. Further, this review focused on cross-sectional studies of HRQoL and inferences about the predictors of long-term outcomes or predictors of changes in quality of life are not possible. Qualitative research and non-peer reviewed papers were excluded from the search which may have limited the evidence collected. In addition, the exclusion of non-English articles may have geographically and ethnically limited the sample of studies reviewed. Finally, due to the sparsity of studies and heterogeneity of methods and measures, a meta-analysis was not possible to quantify the effect of each predictor across the reviewed studies.

## Conclusion

We found significant gaps in understanding predictors of HRQoL in neurodevelopmental conditions, especially outside of autism and ADHD. Cross-condition studies of these predictors are critically needed to enable care models that address shared needs of neurodivergent individuals transcending diagnostic boundaries. Outside of symptoms, our review identified several such need areas that may be associated with HRQoL outcomes, including mental health, social determinants of health, access to care, family context, and positive self-perceptions. Further understanding of HRQoL from the perspective of neurodivergent communities is highly needed.

### Supplementary Information

Below is the link to the electronic supplementary material.

Supplementary file1 (DOCX 16 KB)

Supplementary file2 (DOCX 15 KB)

Supplementary file3 (DOCX 23 KB)

## Abbreviations

ADHD: Attention-deficit/hyperactivity disorder
HRQoL: Health-related quality of life
OCD: Obsessive–compulsive disorder
PedsQL: Pediatric quality of life inventory
PRISMA: Preferred reporting items for systematic reviews and meta-analysis checklist
QoL: Quality of life
SES: Socioeconomic status

## Footnotes

1 In this paper, we will adopt identify-first language (“autistic individuals”) where possible, recognizing that these preferences may vary across the community.
