# Supplementary material for: Predictors of Health-Related Quality of Life in Neurodivergent Children: A Systematic Review
Source: Clin Child Fam Psychol Rev. 2023 Dec 9;27(1):91–129. doi: 10.1007/s10567-023-00462-3 (PMC10920445; doi:10.1007/s10567-023-00462-3)
Supplement: Supplementary file 1 — Supplementary file1 (DOCX 16 KB) [file 10567_2023_462_MOESM1_ESM.docx]

**Supplementary Table 1**

*Search terms used in the review.*

| **Database** | Search Terms |
| --- | --- |
| **MEDLINE/Embase/Psych Info** | 1. "predict*".ab,ti. 2. "determin*".ab,ti. 3. 1 or 2 4. "quality of life".ab,ti. 5. "*qol".ab,ti. 6. 4 or 5 7. ndd.ab,fx,hw,id,kf,kw,mh,ot,ox,sh,sy,ti,tw. 8. "neurodevelopmental disorder$".ab,fx,hw,id,kf,kw,mh,ot,ox,sh,sy,ti,tw. 9. "neurodevelopmental disabilit* ".ab,fx,hw,id,kf,kw,mh,ot,ox,sh,sy,ti,tw. 10. asd.ab,fx,hw,id,kf,kw,mh,ot,ox,sh,sy,ti,tw. 11. "autis*".ab,fx,hw,id,kf,kw,mh,ot,ox,sh,sy,ti,tw. 12. "attention deficit hyperactivity disorder".ab,fx,hw,id,kf,kw,mh,ot,ox,sh,sy,ti,tw. 13. adhd.ab,fx,hw,id,kf,kw,mh,ot,ox,sh,sy,ti,tw. 14. "intellectual disorder$".ab,fx,hw,id,kf,kw,mh,ot,ox,sh,sy,ti,tw. 15. "intellectual disab*".ab,fx,hw,id,kf,kw,mh,ot,ox,sh,sy,ti,tw. 16. "Intellectual Developmental Disorder".ab,fx,hw,id,kf,kw,mh,ot,ox,sh,sy,ti,tw. 17. "Global Developmental Delay".ab,fx,hw,id,kf,kw,mh,ot,ox,sh,sy,ti,tw. 18. "communication disorder$".ab,fx,hw,id,kf,kw,mh,ot,ox,sh,sy,ti,tw. 19. "language disorder$".ab,fx,hw,id,kf,kw,mh,ot,ox,sh,sy,ti,tw. 20. "Speech disorder$".ab,fx,hw,id,kf,kw,mh,ot,ox,sh,sy,ti,tw. 21. "Speech sound disorder$".ab,fx,hw,id,kf,kw,mh,ot,ox,sh,sy,ti,tw. 22. "fluency disorder$".ab,fx,hw,id,kf,kw,mh,ot,ox,sh,sy,ti,tw. 23. "stutter*".ab,fx,hw,id,kf,kw,mh,ot,ox,sh,sy,ti,tw. 24. "learning disorder$".ab,fx,hw,id,kf,kw,mh,ot,ox,sh,sy,ti,tw. 25. "Impairment in reading".ab,fx,hw,id,kf,kw,mh,ot,ox,sh,sy,ti,tw. 26. "Impairment in written expression".ab,fx,hw,id,kf,kw,mh,ot,ox,sh,sy,ti,tw. 27. "Impairment in math*".ab,fx,hw,id,kf,kw,mh,ot,ox,sh,sy,ti,tw. 28. "motor disorder$".ab,fx,hw,id,kf,kw,mh,ot,ox,sh,sy,ti,tw. 29. "developmental coordination disorder$".ab,fx,hw,id,kf,kw,mh,ot,ox,sh,sy,ti,tw. 30. "Stereotypic Movement Disorder".ab,fx,hw,id,kf,kw,mh,ot,ox,sh,sy,ti,tw. 31. "tic disorder$".ab,fx,hw,id,kf,kw,mh,ot,ox,sh,sy,ti,tw. 32. "Tourette's Disorder".ab,fx,hw,id,kf,kw,mh,ot,ox,sh,sy,ti,tw. 33. 7 or 8 or 9 or 10 or 11 or 12 or 13 or 14 or 15 or 16 or 17 or 18 or 19 or 20 or 21 or 22 or 23 or 24 or 25 or 26 or 27 or 28 or 29 or 30 or 31 or 32 34. 3 and 6 and 33 |
| **PubMed** | (predict*[Title/Abstract] OR determin*[Title/Abstract]) AND ("quality of life"[Title/Abstract] OR *qol[Title/Abstract]) AND (ndd[Title/Abstract] OR neurodevelopmental disorder[Title/Abstract] OR neurodevelopmental disabilit*[Title/Abstract] OR asd[Title/Abstract] OR autis*[Title/Abstract] OR attention deficit hyperactivity disorder[Title/Abstract] OR adhd[Title/Abstract] OR intellectual disorder[Title/Abstract] OR "intellectual disab*"[Title/Abstract] OR "Intellectual Developmental Disorder"[Title/Abstract] OR "Global Developmental Delay"[Title/Abstract] OR communication disorder[Title/Abstract] OR language disorder[Title/Abstract] OR Speech disorder[Title/Abstract] OR Speech sound disorder[Title/Abstract] OR fluency disorder[Title/Abstract] OR stutter*[Title/Abstract] OR learning disorder[Title/Abstract] OR "Impairment in reading"[Title/Abstract] OR "Impairment in written expression"[Title/Abstract] OR "Impairment in math*"[Title/Abstract] OR motor disorder[Title/Abstract] OR developmental coordination disorder[Title/Abstract] OR Stereotypic Movement Disorder[Title/Abstract] OR tic disorder[Title/Abstract] OR Tourette's Disorder[Title/Abstract]) |
| **Cochrane** | (predict*[Title/Abstract] OR determin*[Title/Abstract]) AND ("quality of life"[Title/Abstract] OR *qol[Title/Abstract]) AND (ndd[Title/Abstract] OR neurodevelopmental disorder[Title/Abstract] OR neurodevelopmental disabilit*[Title/Abstract] OR  asd[Title/Abstract] OR autis*[Title/Abstract] OR attention deficit hyperactivity disorder[Title/Abstract] OR adhd[Title/Abstract] OR intellectual disorder[Title/Abstract] OR communication disorder[Title/Abstract] OR language disorder[Title/Abstract] OR Speech disorder[Title/Abstract] OR Speech sound disorder[Title/Abstract] OR fluency disorder[Title/Abstract] OR stutter*[Title/Abstract] OR learning disorder[Title/Abstract] OR motor disorder[Title/Abstract] OR developmental coordination disorder[Title/Abstract] OR Stereotypic Movement Disorder[Title/Abstract] OR tic disorder[Title/Abstract] OR Tourette's Disorder[Title/Abstract]) |
