# Supplementary material for: Predictors of Health-Related Quality of Life in Neurodivergent Children: A Systematic Review
Source: Clin Child Fam Psychol Rev. 2023 Dec 9;27(1):91–129. doi: 10.1007/s10567-023-00462-3 (PMC10920445; doi:10.1007/s10567-023-00462-3)
Supplement: Supplementary file 2 — Supplementary file2 (DOCX 15 KB) [file 10567_2023_462_MOESM2_ESM.docx]

**Supplementary Table 2**

*Risk of Bias Questionnaire Adapted from the Cochrane Template.*

| **Question** | **Description** |
| --- | --- |
| **Risk of bias in the selection process for the target population (Was the selection process appropriate)?** | High bias: Participants differ from the population of interest. For example: the study recruits adolescents of mostly of higher socioeconomic status.    Participants should be described in detail (i.e., at least one of demographics, location etc.)  1.High  2.Low  3.Unsure |
| **Risk of bias in describing target population (Was the target population clearly defined)?** | High bias: Inclusion and exclusion criteria are not clearly specified. For example: Study says "Participants with an ADHD-like symptoms were recruited" and provides no further detail.    Sufficient detail should be provided about participants.  1.High  2.Low  3.Unsure |
| **Risk of bias in outcome statistics reporting (Is it clearly stated?)** | High bias: Only a subset of original outcomes measured/analyzed are reported. For example, results that are not significant are deliberately not included in results.    Outcomes should be comprehensively reported, no omission of outcomes mentioned or underreporting should take place.    If RCT:  High bias: Attrition and exclusions were not reported or reasons were not explained, the numbers in each intervention group were not reported (compared with total randomized participants).  1.High  2.Low  3.Unsure |
| **Risk of bias in statistical methods (Are the statistical methods clearly described)** | High bias: statistical approach appropriate was not appropriate or statistical methods poorly described. For example: A study using only correlations to determine the association with an outcome predictor or if statistical analysis are not described in detail. The methods section should be detailed and comprehensive for the reader.  1.High  2.Low  3.Unsure |
| **Other Bias** | State any important concerns about bias not addressed in the other domains in the tool. If questions/entries were pre-specified in the review’s protocol, responses should be provided for each question/entry. |
