# Supplementary material for: Predictors of Health-Related Quality of Life in Neurodivergent Children: A Systematic Review
Source: Clin Child Fam Psychol Rev. 2023 Dec 9;27(1):91–129. doi: 10.1007/s10567-023-00462-3 (PMC10920445; doi:10.1007/s10567-023-00462-3)
Supplement: Supplementary file 3 — Supplementary file3 (DOCX 23 KB) [file 10567_2023_462_MOESM3_ESM.docx]

**Supplementary Table 3**

*Groupings of predictor variables in reviewed studies.*

| **Biology/Physiology Predictors** | |
| --- | --- |
| **Category** | **Variables Merged** |
| **Physical Health/well-being** | No chronic pain  No headaches/migraines  Physical wellbeing |
| **Microcephaly and/or dysmorphology** | Microcephaly and/or dysmorphology |
| **Symptom Predictors** | |
| **ASD Symptoms/traits** | ASD symptom severity  Comorbid ASD  PDD/NOS vs autistic disorder  Calibrated ADOS Severity Score  Screening for autism  Behavioral problem  Autism-related traits |
| **Stutter Symptoms** | Self-assessment scale of speech  Speech Satisfaction score  Stuttered speech frequency  Stuttering severity |
| **Tic disorder symptoms** | Tic disorder type  Tic form  Tic severity  Tic impairment  Premonitory Urge to Tic  Coprophenomena  Motor tics  Phonic/vocal tics  Frequency  Intensity  Complexity  Interference  Number  Coprolalia  Echopraxia  Echolalia |
| **ADHD symptoms** | ADHD Rating Scale: inattentive, hyperactivity, and total score  Cognitive Problem- Inattention  Severity of the ADHD symptoms measured through CPRS: attitude problems, learning problems, psycho-physical problems, impulsivity-hyperactivity problems, anxiety problems  Impact of ADHD  Consequence of ADHD symptoms |
| **Verbal communication skills** | Improvement in language and communication |
| **Executive functioning** | Executive functioning  Executive attentional functioning  Working memory  Cognitive flexibility  Theory of Mind |
| **Behavioural problems** | Frequency and intensity of behavioral and emotional difficulties measured through CBCL total score  Problematic mealtime behaviour |
| **Obsessive compulsive symptoms** | OCD Symptom Severity  Type and extensiveness of OCD symptoms |
| **Schizotypal personality** | Cognitive-Perceptual  Suspiciousness  Interpersonal  No Close Friends  Constricted Affect  Total  Cognitive-Perceptual  Ideas of Reference  Suspiciousness  Magical Thinking  Unusual Perception  Interpersonal  No Close Friends  Constricted Affect  Disorganized  Eccentric Behaviour  Odd Speech |
| **Anxiety** | Anxiety  Social anxiety  Anxiety disorder  Performance anxiety  Anxious arousal  Separation anxiety  Uncertainty anxiety  Physical Anxiety  Harm subscale  Current level of anxiety  Level of sensitivity to regular anxiety |
| **Depression** | Depression  Maladaptive cognitions commonly associated with depression  Automatic negative thoughts (negative self-statements) associated with depression  (Severity of) depressive symptoms  Sadness  Negative effect |
| **Psychological comorbidities** | Anxiety and Depression  Other/ unspecified comorbidities |
| **Gastrointestinal challenges** | Vomiting  Reflux  Diarrhea  Constipation |
| **Medication** | Daily Medication  Medication perceived need  Irregular medication Use  Medication for sleep |
| **Functioning Predictors** | |
| **Adaptive Functioning** | Independence in daily living skill  Adaptive functioning and adaptive behavior measured through VABS: communication, daily living skills, socialization, motor skills |
| **Activities of daily living** | Instrumental Activity of Daily Living  Daily activities (work or school) |
| **General Health Perception Predictors** | |
| **Beliefs/perception about cause** | Parenting  Genes  Brain abnormality  Learning from friends  Schoolwork was too hard  Insufficient efforts  God’s plan  Importance to psychological and environmental causes |
| **Symptom/illness identify** | Identifying as a member of the group  Identity with symptoms and label |
| **Individual Predictors** | |
| **Age** | Age  Grade |
| **Employment status** | Employed  Has income |
| **Weight** | BMI |
| **IQ** | Full-scale IQ  Non-verbal IQ  Cognitive ability  Cognitive functioning |
| **Temperament** | Extraversion/surgency  Effortful control  Orienting sensitivity  Fear  Discomfort  Frustration  Positive affect  High-intensity pleasure  Attentional control  Inhibitory control  Activation control  Neutral perceptual sensitivity  Affective perceptual sensitivity  Associative sensitivity |
| **Coping Strategy** | Acceptance of ADHD  Less emotional reaction/responses  Less distance  Less wishful Thinking  Coping Inventory for Stressful Situations -task oriented (CISS-T)  Coping Inventory for Stressful Situations -avoidance oriented (CISS-A)  Not confronting  Not distancing  Not self-controlling  Seeking social support  Accepting responsibility  No escape-avoidance  Planful problem solving  Positive reappraisal  Task-oriented  Avoidance-oriented  Less emotion-oriented |
| **Self-determination** | Self-determined  Autonomous functioning  Self-regulation  Psychological empowerment  Self-realization |
| **Sexual wellbeing** | Sexual self-esteem  Sexual depression  Sexual worries |
| **Positive self perception** | Self concept  Self esteem  Positive self evaluation |
| **Education** | College or vocational diploma  University degree  Secondary school education |
| **Healthy sleep patterns** | Unchanged sleep schedule during COVID-19  No sleep problems |
| **Physical activity** | Daily Sedentary Behaviour on weekdays  Daily Sedentary Behaviour on weekends  Physical Activity Level during weekdays  Physical Activity Level during weekends  Physical activity 1-2 days a week  Physical activity 3-4 days a week  Physical activity more than 5 days a week  More than 12000 daily steps |
| **Environmental Predictors** | |
| **SES** | Years of seniority on the job  Fathers education  Parents’ education  Maternal schooling  Paternal schooling  Family income  Mother’s job  Father’s job  Number of rooms in the house  Self education level  Personal monthly income  Self degree/diploma |
| **Social assistance** | Social assistance in childhood  Social assistance in adulthood |
| **Leisure activities in the community** | Adequate outdoor activities and entertainment  Participating in community leisure activities |
| **Social support** | Friends  Number of friends  Having a personal friend  Social interaction not worsened by Covid-19  Social support  Regularly visiting friends  Visiting home  Quality of contact with mother  Quality of contact with father  Intimate relationship |
| **Geography** | Living Arrangements  City Dimensions  Urbanity (urban vs. rural) |
| **Access to health care** | Health insurance  Regular access of healthcare  Periodic psychological checkup  Availability of required facilities  Unmet needs  Telehealth |
| **Receiving care** | Receiving medical psychological care |
| **Self-help support group/ self-help organizations** | Previous involvement  Current involvement  Participation  Years participating  Consistency of attendance |
| **Parental age** | Parent age  Mother’s age  Father’s age |
| **Sibling with neurodevelopmental disorder (NDD)** | Sibling with specific learning difficulty (SpLD)  Sibling with ADHD |
| **Poor parental mental health** | Parental mental health  Parent stress  Parent depressive symptoms  Maternal depression  Maternal anxiety  Maternal stress  Salivary cortisol mother  Salivary testosterone Maternal  Paternal depression  Caregiver’s mental health status  Parental stress  Maternal stress  Restriction of social activities for parents |
| **Parenting style** | Feeding strategies  Democratic Parenting Style |
| **Parent physical activity** | Parent Physical Activity during Leisure time  Parent Physical Activity Level during working days |
| **Family functioning** | Harmonious family relationship inharmony family relationship (Lower QoL)  Play at table with parents  Structured and organized family environment  Achievement-oriented family  Positive emotional climate |
| **Family structure** | Family type  Only child  First born  Nuclear |

ADHD: Attention-deficit Hyperactivity Disorder, ASD: Autism Spectrum Disorder, ADOS: Autism Diagnostic Observation Schedule, BMI: body mass index, CBCL: Child Behaviour Checklist, CPRS: Conners’ Parent Rating Scale, IQ: intelligence quotient, OCD: Obsessive Compulsive Disorder, PDD/NOS: Pervasive developmental disorder/Not otherwise specified, SES: Socioeconomic status, VABS: Vineland Adaptive Behavior Scales
